# Effects of meal regularity and snacking frequency on irritable bowel syndrome

**DOI:** 10.3389/fpubh.2025.1675975

**Published:** 2025-10-13

**Authors:** Sarah M. Ajabnoor

**Affiliations:** ^1^Department of Clinical Nutrition, Faculty of Applied Medical Sciences, King Abdulaziz University, Jeddah, Saudi Arabia; ^2^Food, Nutrition and Lifestyle Unit, King Fahd Medical Research Centre, King Abdulaziz University, Jeddah, Saudi Arabia

**Keywords:** irritable bowel syndrome, dietary practices, eating behavior, meal frequency, snack frequency

## Abstract

**Background:**

Dietary practices often trigger irritable bowel syndrome (IBS) symptoms. This study primarily aimed to identify and compare the frequency of different eating behaviors in free-living adults in Saudi Arabia with either self-diagnosed IBS or IBS diagnosed based on Rome IV criteria. The study also examined how irregular eating affects IBS symptom severity.

**Methods:**

This cross-sectional study included 204 adults, 106 (52%) with self-diagnosed IBS and 98 (48%) with Rome IV-defined IBS. All participants completed a questionnaire assessing sociodemographic characteristics, IBS diagnosis (using Rome IV criteria), IBS symptom severity scale (IBS-SSS), and eating patterns.

**Results:**

Irregular eating patterns were similarly reported in both groups. Dietary practices such as not eating meals on a regular schedule and skipping breakfast were reported by approximately 20 and 30% of the participants in each group, respectively. In the regression analysis, frequent snacking was independently associated with lower IBS-SSS, while chewing difficulty, chronic conditions, and type of IBS diagnosis predicted higher severity (adjusted *R*^2^ = 0.260, *p* < 0.001).

**Conclusion:**

Frequent snacking is linked to reduced IBS symptom severity; however, there is a need to consider dietary behaviors alongside comorbid conditions and type of IBS diagnosis in IBS management. Further research into self-diagnosed IBS (a widely prevalent patient group) is required to better understand how these individuals differ from those with a formal diagnosis.

## Introduction

Irritable bowel syndrome (IBS) is considered one of the main reasons for gastroenterology, family medicine, and internal medicine clinic visits (1). IBS is a type of functional bowel disorder in which persistent stomach pain is associated with altered defecation or bowel habits (i.e., constipation, diarrhea, or both) and abdominal bloating symptoms (2). The etiology and pathophysiology of IBS remain unclear. However, while several pathogenic factors have been suggested to increase susceptibility to developing IBS symptoms, they are not necessarily seen in all patients, including genetic risk factors, nervous system dysregulation that alters gut motility, food intolerances, allergies, psychological disturbances, and alteration in the gut microbiome (3, 4).

A recent systematic review and meta-analysis of 57 population-based studies reported that the global prevalence of IBS was 9.2% based on studies that applied the Rome III criteria but 3.8% based on studies that applied the Rome IV criteria (5). IBS was found to be more prevalent in women than men (5). However, the estimated prevalence of IBS varies among countries. In Saudi Arabia, a recent national survey by Bin Abdulrahman et al. reported that the prevalence of IBS across the five geographical regions of Saudi Arabia was 16.4% based on the Rome IV criteria (6). Therefore, the prevalence of IBS in Saudi Arabia is higher but relatively comparable to the global prevalence. Nonetheless, larger epidemiological studies are needed to confirm the national prevalence of IBS in Saudi Arabia.

No specific biomarker currently exists to diagnose IBS. The common diagnostic approach for IBS is a ‘diagnosis of exclusion’, where several unnecessary tests are conducted to rule out other gastrointestinal (GI) conditions. However, an international team of experts on gut-brain interaction disorders developed the Rome criteria, which can be used to diagnose IBS in clinical and research settings (2). It is considered a systematic approach with limited diagnostic tests and careful follow-up.

Research indicates that self-reported or self-diagnosed IBS is considerably more prevalent than cases identified through standardized diagnostic criteria, with self-diagnosed rates often two to three times higher (1, 5, 7–10). Despite not having a formal diagnosis, individuals who identify themselves as having IBS report similar symptom intensity, reduced quality of life, and comparable healthcare usage to those with clinically confirmed IBS (7, 11). Targeting this group helps develop effective dietary interventions and improve resource allocation.

The treatment of IBS is challenging. The available medical treatment options for IBS mainly target symptom reduction, stress reduction, and relaxation. Several drugs have been recommended to manage IBS symptoms associated with dysmotility, visceral hypersensitivity, or psychological issues (12). Another therapeutic option is psychological therapies, such as gut-directed hypnotherapy (12). Strong evidence supports the role of gut-brain interaction as a key factor in advancing future IBS diagnosis and treatment strategies (13). Based on the dietary treatment recommendations for IBS, the first-line therapy should focus on dietary intake and lifestyle assessment, including evaluating alcohol, fat, fiber, spice, and probiotic intake. However, when symptoms persist, a diet lower in low fermentable oligosaccharides, disaccharides, monosaccharides, and polyols (FODMAP) can be considered under the supervision of a dietitian (14).

Food and dietary practices are well-known triggers of IBS symptoms. Previous international population-based studies have identified common dietary triggers and patterns associated with IBS symptoms (15–17). Such dietary practices must be taken into consideration when managing these patients clinically. Several international studies have explored the connection between eating habits and the prevalence of IBS. For instance, previous research has evaluated how the frequency of meals and snacks correlates with IBS occurrence, reported reduced odds of IBS among individuals adhering to regular meal routines (15–17). Despite these efforts, no studies have specifically analyzed the impact of irregular meal timing and snacking frequency on the severity of IBS symptoms. While in Saudi Arabia, most of the available research evidence on IBS is based on prevalence studies that focused on investigating the role of non-dietary related risk factors, such as reduced immunity, psychological factors, genetics, and environmental factors (18). Therefore, this study primarily aimed to identify and compare eating behaviors in individuals in Saudi Arabia with self-diagnosed IBS and those diagnosed with IBS according to the Rome IV criteria Studying self-diagnosed IBS (a widely prevalent patient population) is important due to its impact on public health, workplace productivity, and the healthcare system (11). A secondary aim of this study was to investigate the association between irregular eating patterns and symptom severity in these two groups.

## Methods

### Study design, participants, and data collection

This cross-sectional study included adults subject aged ≥ 18 years residing in different regions in Saudi Arabia and suffering from IBS-related symptoms (i.e., complaining of frequent abdominal pain at least once a week during the last 3 months). While the exclusion criteria involved the following: subjects with no frequent complaints of abdominal discomfort, pregnant women, and subjects who did not consent or who agreed not to participate.

This study was conducted according to the Declaration of Helsinki and was approved by the Research and Ethics Committee of the Faculty of Applied Medical Sciences at King Abdulaziz University (approval no. FAMS-EC2022-06). The first page of the online survey contained a participant information sheet, which included a required question verifying the participant’s consent to participate in this study.

The survey was delivered via the online Survey Monkey platform and distributed between April 2022 and May 2023. Eligible participants were recruited using convenience sampling, where allocated data collectors across Saudi Arabia invited eligible participants via social media platforms such as WhatsApp. Eligible participants were recruited based on the inclusion criteria mentioned earlier, which applied to capture a broad sample of participants experiencing IBS-like symptoms, regardless of whether they had received a formal clinical diagnosis. This approach included both participants with self-diagnosed and Rome IV-defined IBS, thereby enhancing the generalizability of the findings.

The required sample size was estimated using the Epi Info™ software (version 7.2.4.0; US Centers for Disease Control and Prevention, Atlanta, GA, USA). A recent nationwide study in Saudi Arabia reported that the prevalence of IBS was 16.4% based on the Rome IV criteria (6). Therefore, considering this prevalence, a 90% confidence level, a 5% margin of error, and a design effect of 1, the estimated sample size for this study was 170.

### Questionnaire

The questionnaire was provided in Arabic and consisted of four sections comprising 26 questions, which covered four domains including: demographic characteristics (e.g., age, sex, region, education, employment, and medical history of various chronic conditions, including IBS); the Rome IV IBS diagnostic criteria (translated into Arabic), which the author was licensed to use by the Rome Foundation (19, 20); the IBS symptom severity scale (IBS-SSS) (21); and eating patterns.

Self-diagnosis of IBS was based on the following question in the survey: Do you think you have IBS, which is a chronic health condition involving abdominal pain or discomfort associated with altered bowel movement habits like constipation or diarrhea? This mainly reflect the description used routinely by physicians in clinical practice.

All participants (including self-diagnosed IBS and Rome IV-defined IBS groups) completed the IBS-SSS developed by the Rome Foundation, which consists of five questions: abdominal pain frequency (the number of days with pain during the last 10 days multiplied by 10 [0–100]), abdominal pain intensity (rated on a visual analogue scale [VAS] from 0 to 100), abdominal distension (rated on a VAS from 0 to 100), bowel habit dissatisfaction (rated on a VAS from 0 to 100), and life interference (rated on a VAS from 0 to 100). Thus, the final IBS-SSS score ranges from 0 to 500, with higher numbers denoting more severe symptoms (21).

The questions about eating patterns were designed using information from an earlier, validated survey that examined the association between dietary patterns and IBS prevalence (16). The questionnaire was tested on a random sample for clarity along with a panel of three experts who evaluated the questionnaire’s content validity; it was adjusted by the investigator based on the recommendations and input received.

### Outcome variable

The primary outcome of interest was the IBS-SSS, a continuous measure that quantifies the severity of IBS-related symptoms, including abdominal pain frequency and intensity, bloating, dissatisfaction with bowel habits, and interference with daily activities. While the diagnosis of IBS was based on the Rome IV criteria, the IBS-SSS served as the dependent variable in this study.

### Independent variables

The independent variables encompassed a range of eating behavior indicators—such as the number of main meals consumed per day, frequency of snacking, meal timing regularity, weekly breakfast consumption, water intake during meals, and the ability to chew food properly. Additionally, sociodemographic and health-related characteristics were included: age, gender, geographic region, educational attainment, employment status, presence of chronic conditions, and Rome IV-defined IBS status.

### Statistical analysis

The data were analyzed using the Statistical Package for Social Sciences (version 25; IBM Corp., Armonk, NY, USA). The categorical variables are presented as frequencies (percentages), and continuous variables are presented as the mean ± standard deviation. The normality of the continuous variables’ data distribution was evaluated using a histogram and the Shapiro–Wilk test. Eating behaviors were compared by IBS type and demographic characteristics using the chi-square and Fisher’s exact tests. The severity of IBS symptoms (as measured by the IBS-SSS) was compared by the type of IBS diagnosis using the Mann–Whitney U. A two-sided *p*-value of <0.05 was considered statistically significant.

To explore the relationship between eating behaviors and IBS-SSS (outcome variable), multiple linear regression analyses were conducted. The first model (Model 1) included only dietary behavior variables: number of main meals per day, frequency of daily snacking, regularity of meal patterns, frequency of breakfast consumption per week, water intake during meals, and chewing ability. Model 2 extended the analysis by adjusting for sociodemographic factors as well as clinical variables, including the presence of chronic conditions and type of IBS diagnosis.

## Results

**Figure 1:**
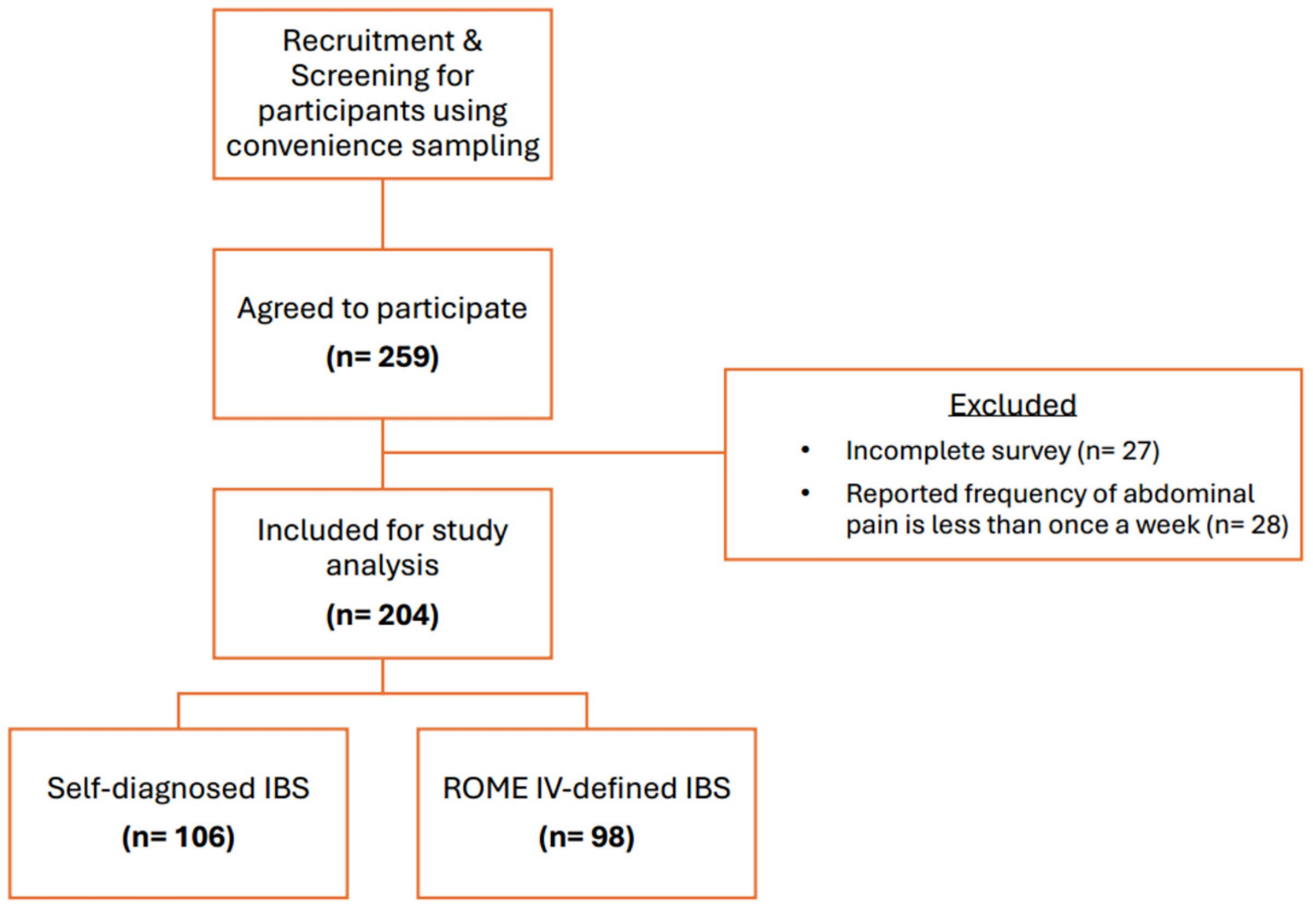
Flowchart of study recruitment. Incomplete surveys: refers to questionnaires with missing key variables.

A total of 259 individuals agreed to participate in the study, of which 204 were considered eligible for inclusion. Of the eligible participants, 106 (52%) self-diagnosed IBS symptoms and 98 (48%) were diagnosed with IBS based on the Rome IV criteria questionnaire. The flowchart of the study recruitment process is illustrated in Figure 1.

Most participants were female (86.8%). The number of reported medical complaints was higher in the Rome IV IBS group than in the self-diagnosed IBS group. The demographic characteristics of both groups are presented in Table 1.

**Table 1:** Demographic characteristics of the study participants.

Characteristics	Self-diagnosed IBS (*n* = 106)	Rome IV-defined IBS (*n* = 98)	All participants (*n* = 204)	*p*-value
	*n* (%)	*n* (%)	*n* (%)	
Age group				
18–29 years	42 (39.6%)	45 (45.9%)	87 (42.6%)	0.658
30–39 years	25 (23.6%)	23 (23.5%)	48 (23.5%)	
40–49 years	27 (25.5%)	18 (18.4%)	45 (22.1%)	
50–59 years	9 (8.5%)	7 (7.1%)	16 (7.8%)	
≥60 years	3 (2.8%)	5 (5.10%)	8 (3.9%)	
Sex				
Female	92 (86.80%)	85 (86.70%)	177 (86.8%)	0.990
Male	14 (13.20%)	13 (13.30%)	27 (13.2%)	
Region				
Western region	73 (68.9%)	68 (69.4%)	141 (69.1%)	0.533
Eastern region	9 (8.5%)	4 (4.1%)	13 (6.4%)	
Central region	15 (14.2%)	20 (20.4%)	35 (17.2%)	
Southern region	4 (3.8%)	3 (3.1%)	7 (3.4%)	
Northern region	5 (4.7%)	3 (3.1%)	8 (3.9%)	
Educational level				
Less than high school	2 (1.9%)	2 (2.0%)	4 (2.0%)	0.757
High school	13 (12.3%)	10 (10.2%)	23 (11.3%)	
Diploma	4 (3.8%)	3 (3.1%)	7 (3.4%)	
Bachelor’s	70 (66.0%)	73 (74.5%)	143 (70.1%)	
Higher education degree	17 (16%)	10 (10.2%)	27 (13.2%)	
Employment status				
Student	21 (19.8%)	21 (21.4%)	42 (20.6%)	0.826
Employed	48 (45.3%)	49 (50%)	97 (47.5%)	
Unemployed	29 (27.4%)	24 (24.5%)	53 (26%)	
Retired	8 (7.5%)	4 (4.1%)	12 (5.9%)	
Medical history of conditions diagnosed by a physician^†^				
None	40 (37.7%)	21 (21.4%)	61 (29.9%)	0.011^*^
Diabetes	9 (8.5%)	11 (11.2%)	20 (9.8%)	0.512
Hypertension	6 (5.7%)	12 (12.2%)	18 (8.8%)	0.098
Dyslipidaemia	0 (0%)	5 (5.1%)	5 (2.5%)	0.019^*^
Thyroid problems	12 (11.3%)	11 (11.2%)	23 (11.3%)	0.983
Maldigestion	9 (8.5%)	19 (19.4%)	28 (13.7%)	0.024^*^
GERD	1 (0.9%)	3 (3.1%)	4 (2.0%)	0.276
Celiac disease	0 (0%)	0 (0%)	0 (0%)	-
Lactose intolerance	5 (4.7%)	13 (13.3%)	18 (8.8%)	0.032^*^
Inflammatory bowel disease	0 (0%)	6 (6.1%)	6 (2.9%)	0.010^*^
Chronic constipation	16 (15.1%)	28 (28.6%)	44 (21.6%)	0.019^*^
History of GI surgery	2 (1.9%)	4 (4.1%)	6 (2.9%)	0.354
Others	3 (2.8%)	8 (8.2%)	11 (5.4%)	0.092

Data are presented as numbers and percentages.

^†^Total percentage may exceed 100% because participants were allowed to choose more than one option (multiple-response items).

The frequency of most eating behaviors did not differ significantly between the two groups. They showed similar trends in the regularity of eating meals and breakfast intake. The proportion who do not eat meals on a regular schedule was 20% in each group, while the proportion who do not eat breakfast or only eat it once a week was 34% in the self-diagnosed IBS group and 31.6% in the Rome IV IBS group. However, the ability to chew the food differed significantly between groups (*p* < 0.05). The Rome IV IBS group was more likely (15.3%) to prefer soft and pureed food for chewing than the self-diagnosed IBS (3.8%). The eating behaviors of both groups are compared in Table 2.

**Table 2:** Frequency of eating behaviors in patients with self-diagnosed IBS and Rome IV-defined IBS.

Eating behaviors	Self-diagnosed IBS (*n* = 106)	Rome IV-defined IBS (*n* = 98)	All participants (*n* = 204)	*p*-value
	*n* (%)	*n* (%)	*n* (%)	
Frequency of daily meals				
1 meal/day	19 (17.9%)	12 (12.2%)	31 (15.2%)	0.493
2 meals/day	55 (51.9%)	52 (53.1%)	107 (52.5%)	
3 meals/day	32 (30.2%)	34 (34.7%)	66 (32.4%)	
Frequency of daily snacks				
No snacks	18 (17%)	18 (18.4%)	36 (17.6%)	0.869
1–2 snacks/day	80 (75.5%)	71 (72.4%)	151 (74%)	
≥ 3 snacks/day	8 (7.5%)	9 (9.2%)	17 (8.3%)	
Eating meals on a regular schedule				
Never	22 (20.8%)	20 (20.4%)	42 (20.6%)	0.428
Sometimes	57 (53.6%)	49 (50%)	106 (52%)	
Often	20 (18.9%)	26 (26.5%)	46 (22.5%)	
Always	7 (6.6%)	3 (3.1%)	10 (4.9%)	
Regularity of breakfast intake				
Never or once a week	36 (34%)	31 (31.6%)	67 (32.8%)	0.541
2–4 times/week	36 (34%)	27 (27.6%)	63 (30.9%)	
5–6 times/week	9 (8.5%)	13 (13.3%)	22 (10.8%)	
Everyday	25 (23.6%)	27 (27.6%)	52 (25.5%)	
Drinking water with meals				
Never	12 (11.3%)	11 (11.2%)	23 (11.3%)	0.313
Sometimes	35 (33%)	38 (38.8%)	73 (35.8%)	
Often	36 (34%)	22 (22.4%)	58 (28.4%)	
Always	23 (21.7%)	27 (27.6%)	50 (24.5%)	
Ability to chew the food				
No problem	99 (93.4%)	79 (80.6%)	178 (87.3%)	0.011^*^
Only soft and puree food	4 (3.8%)	15 (15.3%)	19 (9.3%)	
Cannot chew the food	3 (2.8%)	4 (4.10%)	7 (3.4%)	

Data are presented as numbers and percentages.

^*^p-value < 0.05.

**Figure 2:**
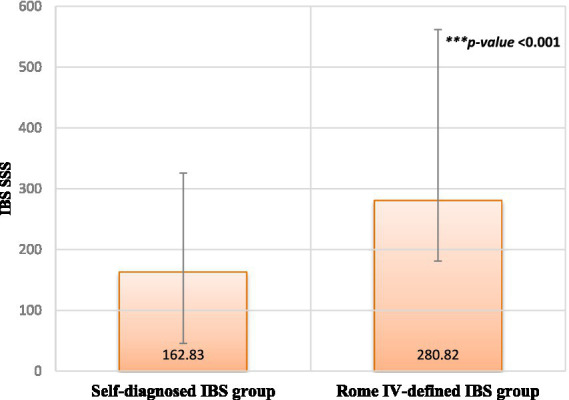
IBS symptoms severity score (IBS-SSS).

Figure 2 presents the difference in IBS-SSS between the two groups. Participants diagnosed with IBS based on the Rome IV criteria exhibited significantly higher severity scores than individuals who self-diagnosed as having IBS (*p* < 0.001).

Multiple linear regression analyses were performed to assess the relationship between eating behaviors and IBS symptom severity (IBS-SSS). In the unadjusted model, which included only eating behavior variables, the overall model demonstrated a modest fit (adjusted *R*^2^ = 0.039, *p* < 0.05). Among the predictors, snack frequency was significantly and inversely associated with IBS-SSS (*β* = −0.189, *p* < 0.01). Conversely, chewing difficulty was positively associated with symptom severity (*β* = 0.149, *p* < 0.05). No significant associations were found for other eating behavior variables (Table 3).

**Table 3:** Multiple linear regression analysis of eating behaviors and IBS symptom severity (IBS-SSS) (*n* = 204).

	Model 1 (Unadjusted) Outcome variable: IBS-SSS			Model 2 (Fully adjusted) Outcome variable: IBS-SSS		
	*R*	*R* ^2^	Adj. *R*^2^	*R*	*R* ^2^	Adj. *R*^2^
Model statistics	0.260	0.068	0.039	0.554	0.307	0.260
Dependent variable	*β*		*p*-value	*β*		*p*-value
Frequency of daily meals	0.100		0.217	0.100		0.170
Frequency of daily snacks^$^	−0.189		0.008^**^	−0.198		0.002^**^
Eating meals on a regular schedule^$^	−0.074		0.355	−0.079		0.267
Frequency of weekly breakfast	−0.038		0.635	−0.070		0.335
Drinking water with meals^$^	0.062		0.377	0.062		0.329
Ability to chew the food^$^	0.149		0.037^*^	0.071		0.263
Chronic conditions^$^	–		–	0.139		0.037^*^
Type of IBS diagnosis^$^	–		–	0.441		<0.001^***^

*β*, standardized regression coefficient.

Model 2: adjusted for sociodemographic covariates (i.e., age, sex, region, education, employment), plus chronic conditions and type of IBS diagnosis.

^$^Reference groups and coding for categorical predictors: Frequency of daily snacks: 0 = No snacks (Ref), 1 = 1–2 snacks/day, 2 = 3 ≥ snacks/day; Eating meals on a regular schedule: 0 = Never (Ref), 1 = Sometimes, 2 = Often, 3 = Always; Drinking water with meals: 0 = Never (Ref), 1 = Sometimes, 2 = Often, 3 = Always; Ability to chew the food: Chronic conditions: 0 = None (Ref), 1 = Yes; Type of IBS diagnosis: 0 = Self-diagnosed IBS (Ref), 1 = Rome IV-defined IBS.

*p-value < 0.05.

**p-value < 0.01.

**p-value < 0.001.

In the adjusted model, which incorporated eating behaviors along with sociodemographic characteristics (i.e., age, gender, region, education, and employment), presence of chronic conditions, and type of IBS diagnosis, the model fit improved with adjusted *R*^2^ = 0.260 (*p* < 0.001). Snack frequency remained a significant negative predictor of symptom severity (*β* = −0.198, *p* < 0.01), whereas the association with chewing difficulty was no longer statistically significant. The presence of a chronic health condition (*β* = 0.139, *p* < 0.05) and type of IBS diagnosis (*β* = 0.441, *p* < 0.001) were both associated with greater symptom severity score.

## Discussion

The present study primarily aimed to identify and compare the frequency of different factors related to eating behaviors in individuals in Saudi Arabia with self-diagnosed IBS and those diagnosed with IBS according to the Rome IV criteria. Its secondary aim was to investigate the association between irregular eating patterns and symptom severity in these two groups. According to the study findings, the frequency of most eating behaviors did not differ significantly between those with self-diagnosed IBS symptoms and those with IBS diagnosed based on the Rome IV criteria, except for the ability to chew food. The Rome IV IBS group was more likely to prefer soft and pureed food for chewing. Dietary practices such as not eating meals on a regular schedule and skipping breakfast were reported by approximately 20 and 30% of participants in each group. Additionally, in the regression analysis, frequent snacking was linked to reduced severity of IBS symptoms, whereas difficulty chewing was associated with greater IBS-SSS. After adjusting for sociodemographic and clinical variables, snack frequency, chronic health conditions, and type of IBS diagnosis were identified as independent predictors of symptom severity.

In this study, most investigated eating behaviors showed no significant differences between individuals with self-diagnosed IBS and those meeting Rome IV criteria, indicating that overall dietary habits may be consistent across groups regardless of their diagnostic classification. Self-diagnosed IBS is widespread and has a notable effect on quality of life, healthcare utilization, and work performance (1, 7). Therefore, individuals with self-diagnosed IBS would possibly adjust their eating habits to alleviate symptoms, resulting in dietary patterns that is relatively similar to those meeting the Rome IV diagnostic criteria. Nevertheless, the key exception was chewing ability; participants with Rome IV-diagnosed IBS were more likely to rely on soft or pureed foods. Dietary preference to consume soft and pureed foods might mitigate IBS symptoms. However, evidence is unclear about the importance of such a recommendation. Significant reductions in IBS symptoms, such as diarrhea, bloating, gas, and abdominal pain, were observed with a starch and sucrose reduced diet in a randomized clinical trial (22). A traditional diet advice given to patients with IBS is to limit the consumption of ‘resistant starch’, which is common in processed or precooked foods and is defined as starch that enters the colon undigested after resisting digestion in the small intestine (23). The American College of Gastroenterology recommends using soluble rather than insoluble fiber to address IBS symptoms (12). In the GI tract, dietary fiber has various unclear mechanisms impacting the gut flora, metabolism, transit time, stool consistency, and absorption of bile acid (12). The current evidence only supports the impact of dietary changes in the management of IBS, while it does not specifically address how soft and pureed foods affect IBS symptoms. Further studies are needed to assess the role of chewing ability and food texture in IBS.

In this study, about 20% of participants skipped regular meals, and 30% frequently skipped breakfast. Studies have shown that irregular eating patterns and skipping meals are common among patients with IBS (15, 24, 25). These studies linked meal regularity with reduced IBS prevalence (15, 24, 25), while the present study characterized the frequency of different eating behaviors in individuals with IBS (either self-diagnosed or Rome IV-diagnosed IBS). The disruption of regular eating patterns can exacerbate IBS symptoms since it may lead to dysregulation of the gut-brain axis and irregular bowel movements, further contributing to discomfort and pain. Consuming a breakfast high in protein and fiber may moderate the link between the frequency of consuming breakfast and IBS risk, even though the exact cause of this association remains unknown (25). Nonetheless, further cohort or interventional research is required to investigate the possible causal relationship between eating breakfast frequently and IBS. Moreover, some studies have highlighted that orthorexia and disordered eating symptoms are common in patients with IBS, especially those with severe GI symptoms and who are more stressed and anxious (26, 27). Therefore, eating disorders should be considered when assessing eating patterns in patients with IBS.

Based on the regression analysis (unadjusted model), only snack frequency and chewing ability showed significant association with IBS-SSS. Frequent snacking was linked with reduced symptom severity, while chewing difficulties were associated with more severe symptoms. These results contrast with previous research that found no significant relationship between snack frequency and IBS risk (15, 28). However, frequent snacking might help manage IBS symptoms by mechanisms such as, delaying gastric emptying and minimizing post-meal bloating. Moreover, the carbohydrates composition found in snacks could impact gut microbiota and glycaemic response, possibly affecting fermentation processes and how symptoms are perceived (29). Future mechanistic studies should explore the impact of snacking frequency on IBS. According to the fully adjusted model, the negative association between snack frequency and symptom severity remained significant, while the impact of chewing ability and other eating behaviors lost significance, suggesting that their effects may be due to confounding factors. Notably, the presence of chronic conditions and the diagnosis of Rome IV–based IBS were the strong predictors of increased symptom severity. IBS is often linked with several chronic health conditions, which can intensify symptoms and lower quality of life, thus, effective treatment strategies should take into account both IBS and its related comorbidities (30). Regarding IBS diagnosis, the Rome IV criteria are considered very restrictive, emphasizing frequent and intense abdominal pain (31). As a result, the criteria will identify a more uniform group of individuals who tend to experience more severe symptoms (31), which helps explain why Rome IV-diagnosed IBS in the present study is typically more severe than self-diagnosed IBS. While diet impacts IBS symptoms, clinical factors play a larger role and warrant further exploration.

The present study appears to be the first to assess the frequency of eating behaviors and its impact on symptoms severity in Saudi adults with IBS. This study has several limitations that should be acknowledged. First, this study did not stratify between IBS with diarrhea (IBS-D) and IBS with constipation (IBS-C), which are managed differently. According to one observational study, patients with IBS-D tend to consume more healthy plant-based foods and had lower microbial diversity (32). The current available evidence is inconclusive regarding the impact of snacking on the different IBS subtypes. Second, using a convenience sampling method may limit the study’s representativeness, as the self-selected participants might not accurately reflect the wider IBS population. Thirdly, the cross-sectional design prevented conclusions from being drawn about the casual association between eating behaviors and IBS. Also, reliance on self-reported dietary patterns and symptoms may be subject to recall and reporting bias. Another limitation is the limited exploration of possible confounding factors like psychological stress, physical activity, and medication use, which could affect both eating habits and the severity of IBS symptoms.

## Conclusion

This study emphasizes that regular eating and frequent snacking can help alleviate IBS symptoms. Though this cross-sectional association should be interpreted with caution, as causal inferences cannot be made. Also, the study findings highlight the need to consider dietary behaviors alongside comorbid conditions and type of IBS diagnosis in managing IBS symptoms. Further longitudinal and intervention studies are needed to determine if structured snacking approaches could support symptom management when included in dietary recommendations. In addition, the comparative approach utilized in this study provides a better understanding of eating patterns (i.e., similarities and differences) among individuals with self-diagnosed IBS and patients diagnosed with IBS according to Rome IV criteria. Further research into self-diagnosed IBS is still required, particularly to better understand how these individuals differ from those with a formal diagnosis. Self-diagnosis should not replace medical evaluation, and healthcare professionals should be encouraged to educate patients on the importance of proper diagnosis to rule out other conditions.

## Data Availability

The raw data supporting the conclusions of this article will be made available by the authors, without undue reservation.
